# Molecular Analysis of Emerging MT27 Macrolide-Resistant Bordetella pertussis, Kobe, Japan, 2025

**DOI:** 10.3201/eid3201.250890

**Published:** 2026-01

**Authors:** Shoko Komatsu, Noriko Nakanishi, Kousaku Matsubara, Yuui Inenaga, Masayuki Hori, Kiyo Shiotani, Rumi Morimoto, Chie Nantani, Yuki Muneta, Nobuya Kusunoki

**Affiliations:** Kobe Institute of Health, Kobe, Japan (S. Komatsu, N. Nakanishi); Kobe City Nishi-Kobe Medical Center, Kobe (K. Matsubara, Y. Inenaga, M. Hori); Public Health Center of Kobe City, Kobe (K. Shiotani, R. Morimoto, C. Nantani, Y. Muneta, N. Kusunoki)

**Keywords:** Bordetella pertussis, bacteria, antimicrobial resistance, macrolide-resistant Bordetella pertussis, MRBP, MT27, prn150, Japan

## Abstract

We report the emergence and spread of multilocus variable-number tandem-repeat analysis type 27 (MT-27) macrolide-resistant *Bordetella pertussis* (MRBP) in Kobe, Japan, in 2025. Whole-genome sequencing revealed that MT27-MRBP did not originate from the widely circulating MT27 macrolide-sensitive *B. pertussis* in Japan but was closely related to MRBP in China.

*Bordetella pertussis*, a gram-negative, pathogenic bacterium of the genus *Bordetella*, is the causative agent of contagious respiratory illness and whooping cough (pertussis). Diphtheria-pertussis-tetanus (DPT) combination vaccines have substantially reduced pertussis-related illness and deaths, especially among infants (*1*). Macrolides represent mostly natural polyketide-class products containing a large macrocyclic lactone ring with potential attachment groups (e.g., deoxy sugars), with antibiotic or antifungal activities. Macrolides are popular pharmaceutical drugs, frequently used for pertussis treatment and prevention. Macrolide-resistant *B. pertussis* (MRBP), characterized by the A2047G mutation in a region critical for macrolide binding to the 23S rRNA gene, has recently emerged and spread worldwide (*2*). In China, the predominant MRBP genetic lineage has shifted in the pertussis toxin promoter region (*ptxP*) allele type from *ptxP1* to *ptxP3*, and the prevalence of the *ptxP3*-carrying multilocus variable-number tandem-repeat analysis type (MT) 28 MRBP strain has increased rapidly (*3*).

In Japan, pertussis notifications, which decreased during the COVID-19 pandemic, have significantly increased since 2024 (*4*). MRBP was first documented in 2018 during the first isolation of *ptxP1*-MT195-MRBP (*5*). More recently, *ptxP3*-MRBP strains isolated from Tokyo and Okinawa have been described, demonstrating close genetic relation to the China strains (*6*,*7*).

MT27 is a single-locus MT28 variant, and this genotype, carrying the virulence-associated alleles *ptxP3*/*ptxA1*/*prn2*/*fim3-1*, became predominant among macrolide-susceptible *B. pertussis* (MSBP) strains in various countries, including Japan (*8*–*10*). In contrast, to date, just 1 MT27-MRBP strain has been reported in China in 2017 (*8*); no cases have been identified outside of China. In this study, we report 5 MT27-MRBP strains isolated during February–March 2025 from children with pertussis in 1 hospital and 2 private clinics in Kobe, Japan (Table). To investigate the molecular epidemiologic characteristics of these 5 MT27-MRBP isolates, we compared them to Japan MT27-MSBP strains isolated during 2010–2025, including 10 isolates from Kobe, and MRBP strains from China (*3*,*6*–*8*,*10*). This study was approved by the Kobe City Review Board (approval no. SenR3-10).

**Table:** Clinical features and microbiological profiles of 15 MT27-*Bordetella pertussis* isolates in Kobe, Japan, January 2013–March 2025*

Strain ID	Collection date	Patients		Macrolide susceptibility	Genotyping of virulence-related genes				
		Age/sex	Vaccination (no. doses)		*ptxP*	*ptxA*	*fhaB*	*fim3*	*prn*
KBP0005	2013 Mar 25	1 y/M	DPT (3)	Susceptible	3	1	1	1	2
KBP0006	2015 Apr 21	4 mo/F	NA	Susceptible	3	1	1	1	2
KBP0007	2016 Feb 25	8 mo/M	Unvaccinated	Susceptible	3	1	1	1	2
KBP0009	2019 Sep 24	1 mo/F	Unvaccinated	Susceptible	3	1	1	1	2
KBP0010	2024 Jun 24	1 mo/F	Unvaccinated	Susceptible	3	1	1	1	2
KBP0011	2024 Jun 24	35 y/M	DPT (4)	Susceptible	3	1	1	1	2
KBP0014	2025 Jan 25	8 y/F	DPT-IPV (4)	Susceptible	3	1	1	1	2
KBP0016	2025 Feb 3	10 y/F	DPT-IPV (4)	Resistant	3	1	1	1	150
KBP0017	2025 Feb 12	2 mo/M	DPT-IPV (1)	Resistant	3	1	1	1	150
KBP0018	2025 Feb 13	12 y/M	DPT-IPV (4)	Susceptible	3	1	1	1	2
KBP0019	2025 Feb 18	9 y/F	DPT-IPV (4)	Susceptible	3	1	1	1	2
KBP0020	2025 Feb 20	10 y/F	DPT-IPV (4)	Resistant	3	1	1	1	150
KBP0025	2025 Mar 7	12 y/F	DPT-IPV (4)	Susceptible	3	1	1	1	2
KBP0026	2025 Mar 7	12 y/F	NA	Resistant	3	1	1	1	150
KBP0028	2025 Mar 12	12 y/F	NA	Resistant	3	1	1	1	150

*DPT, diphtheria-pertussis-tetanus; IPV, inactivated polio vaccine; MT, multilocus variable-number tandem-repeat analysis type; NA, not available.

We collected 9 MT27 strains from patients 2 months through 12 years of age during January–March 2025 (Table). All 5 MT27-MRBP strains harbored the A2047G mutation in the 23S rRNA and exhibited MICs of >256 μg/mL for erythromycin, clarithromycin, and azithromycin. All MT27-MRBP–infected patients recovered without any sequelae. We used the BIGSdb-Pasteur platform (https://bigsdb.pasteur.fr/bordetella) to identify the MT27-MRBP virulence genotype, which yielded identical results for all strains: *ptxP3*/*ptxA1*/*fhaB1*/*fim3–1*/*prn150*. Among the 5 virulence-related genes, we observed a difference in the *prn* allele between the MT27-MSBP and MT27-MRBP strains isolated in Kobe (i.e., *prn2* in MSBP and *prn150* in MRBP) (Table). Of note, *prn150* was identical to the allele in the globally prevalent MT28-MRBP strains (*3*).

To determine genetic relatedness, we performed phylogenetic analyses using whole-genome sequences of 6 MT27-MSBP isolates obtained in Kobe since 2013 (Table) and other publicly available genomes (Appendix Table). Our single-nucleotide variant–based phylogenetic analysis revealed that the 5 MT27-MRBP strains clustered within the *prn150* lineage, which is genetically closely related to the MRBP strain from China and that clonal population (Figure). Furthermore, MT27-MRBP strains in Kobe were genetically distinct from the China MT27-MRBP (GenBank accession no. SRR16306222), as well as from the Japan MRBP strains, BP636 (GenBank accession no. DRR631445) in Tokyo and OkiPb01308 and OkiPb01309 (National Center for Biotechnology Information BioProject accession no. PRJDB20292) in Okinawa (Figure). The identification of genetically divergent strains across 3 geographically separated regions of Japan suggests multiple, epidemiologically independent introductions. In contrast, MT27-MSBP strains KBP0014, KBP0018, KBP0019, and KBP0025, isolated in 2025, belonged to a clade of currently prevalent strains in Japan. Taken together, our results suggest that MT27-MRBP does not originate from the currently circulating MT27-MSBP in Japan but could have been potentially introduced from China. Finally, 5 MT27-MRBP–infected patients resided in 3 different wards with no apparent temporal links, suggesting that this newly emergent strain might be spreading latently in Kobe.

**Figure:**
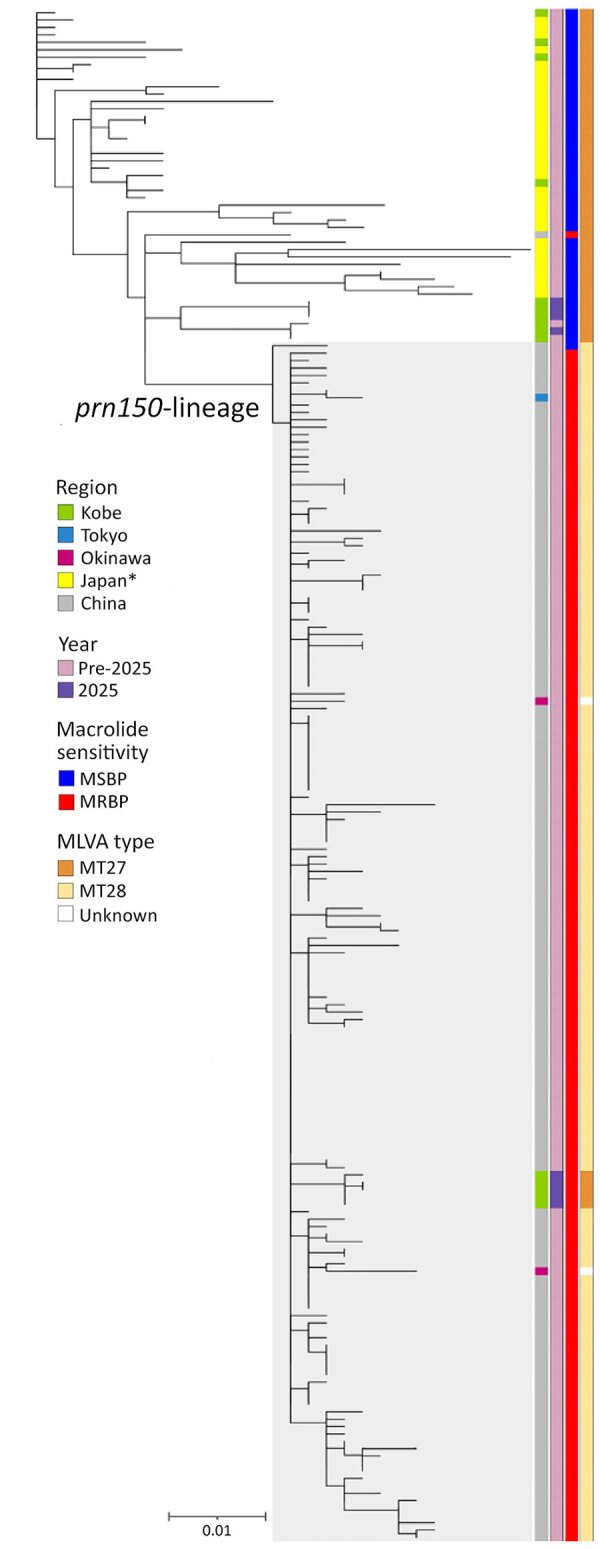
Phylogenetic tree based on single-nucleotide variants, showing 15 MT27 *Bordetella pertussis* strains isolated in Kobe (green); 37 strains from Japan (yellow), including Tokyo (blue) and Okinawa (magenta); and 155 strains from China (gray) in study of emerging MT27 MRBP*,* Kobe, Japan, 2025. Scale bar indicates number of substitutions per site. *Excluding regions previously listed. MRBP, macrolide-resistant *B. pertussis*; MSBP, macrolide-sensitive *B. pertussis*; MLVA, multilocus variable-number tandem-repeat analysis; MT, MLVA type.

In conclusion, we identified distinct genetic differences between the MT27-MSBP and MT27-MRBP strains collected during January–March 2025 in Kobe. Our study suggests that MT27-MRBP strains closely related to the China MRBP strains have emerged and spread in Kobe, Japan.

Appendix Additional information about molecular analysis of emerging MT27 macrolide-resistant *Bordetella pertussis,* Kobe, Japan, 2025.
